# The Association Between Coffee Consumption and Local Anesthesia Failure: Social Beliefs and Scientific Evidence

**DOI:** 10.7759/cureus.7820

**Published:** 2020-04-24

**Authors:** Sangeetha Premnath, Ghadah Alalshaikh, Randa Alfotawi, Manju Philip

**Affiliations:** 1 Department of Oral and Maxillofacial Surgery, College of Dentistry, King Saud University, Riyadh, SAU

**Keywords:** coffee consumption, local anaesthesia, failure, caffeine

## Abstract

Introduction

In our clinical practice, we have encountered patients who reported the failure of local anesthesia due to excessive coffee consumption and required higher-than-normal doses of local anesthesia. Therefore, our study aimed to assess the awareness and knowledge of coffee consumption, its effect on local anesthesia, and the available scientific evidence among the public, patients, and clinicians in dental practice.

Material and Methods

A cross-sectional survey with two sets of questionnaires was designed based on the Likert scale. A 5-point scale was used to assess agreement and frequency. Yes/no and open-ended questions were used for the assessment. Questionnaires were distributed among the clinicians, patients, and the public. Data were analyzed with descriptive linear statistics.

Results

Of the 430 responses provided by patients and the general public, more than 40% believed that the local anesthetic failure was caused by excessive coffee consumption. Among the 235 responses provided by the clinicians, 65% of the clinicians reported encountering patients with local anesthesia failure and believed it could be due to excessive coffee consumption. However, only 9% of the clinicians were aware of scientific evidence regarding the effect of coffee consumption on local anesthesia failure.

Conclusion

Surprisingly, the majority of clinicians believed that caffeine had an effect on the reduction of local anesthesia; however, only a few of them had scientific knowledge. The available scientific evidence relates that caffeine can influence cognitive performance by increasing alertness, as well as sleep deprivation causing stress and anxiety, which partially explains the local anesthetic failure among coffee consumers. Therefore, a stress reduction protocol should be a routine daily practice for a dentist to reduce the failure rate of local anesthesia.

## Introduction

In Saudi Arabia, caffeine is commonly consumed in the form of Arabic coffee, which has become a crucial part of Arab social life [1]. A coffee consumption culture facilitates an image of pride, hospitality, and liberality for the Arabs [1]. In our clinical practice at the College of Dentistry, King Saud University (KSU), we have encountered patients who reported that they often failed to experience numbness after local anesthesia administration due to excessive consumption of coffee and requested higher-than-normal doses of local anesthesia during dental treatment. These observations led us to investigate if there is a relationship between coffee consumption and the failure of local anesthesia. 

One of the main active ingredients of coffee is caffeine, which is affected by the type of coffee bean, roasting style, the method of preparing coffee, and the serving size. The degree of roasting of the coffee beans is inversely proportional to the amount of caffeine and water content in the beans. Arabic coffee (Gahwa Arabi) is brown in color or closer to dark yellow when it less roasted and hence contains more caffeine and water [2]. Roasted coffee is a complex blend of various bioactive compounds with potentially therapeutic antioxidant, anti-inflammatory, antifibrotic, or anticancer effects that may be responsible for recently reported epidemiological associations [1, 3]. However, previous studies aiming to determine whether coffee consumption has beneficial or harmful effects on health have arrived at mixed conclusions [3]. 

Caffeine, which is the chief alkaloid present in coffee, tea, and energy drinks, is one of the most consumed psychoactive substances worldwide [1, 4-5]. It influences various parts of the central nervous system and has an impact on cognitive performance, improves memory, changes the state of mind, and increases alertness [6-7]. Additionally, it has a negative effect on sleep quality, and excessive caffeine intake has been associated with anxiety, headaches, nausea, and restlessness [6-7]. 

Caffeine mainly acts on the central nervous system by inhibiting adenosine receptors, thereby regulating neurotransmitter release [4-5]. Caffeine can oppose the effects of adenosine by acting as an antagonist of adenosine receptors A1 and A2A. It increases neuronal activity by preventing the binding of adenosine to its receptors and has a downstream stimulatory impact on the neurons [4-5, 8]. It also plays an important role in pain modulation through its action on adenosine receptors that are involved in nociception [5]. As a result, it has been used as an adjuvant analgesic to relieve pain [3-4, 9]. 

Local anesthesia is routinely used for various procedures in dental clinical practice, and varieties of local anesthetic agents, as well as anesthetic techniques, have been used to perform pain-free procedures. However, clinicians often fail to obtain adequate anesthesia during inferior alveolar nerve blocks [10]. In some cases, patients may also report experiencing inadequate numbness for routine dental procedures. The published studies on local anesthetic efficacy have reported less than 100% success [10-11]. Local anesthetics prevent the generation and conduction of nerve impulses. Many theories have been put forth to explain the mechanism of action of local anesthetics, which include acetylcholine activity, calcium displacement, surface charge, membrane expansion, and specific receptor theories [8, 12-13]. 

Although to our knowledge, no previous report has directly linked coffee consumption to failure of local anesthesia, various factors have been reported to contribute to the failure of local anesthesia. These may include patient-related factors, such as anatomical, pathological, and psychological factors or operator-dependent factors, like the choice of technique and the amount of solution used [10-12]. 

This study aimed to assess the awareness and knowledge of coffee consumption and its effect on local anesthesia among the public, patients, and clinicians in dental practice in the city of Riyadh. Additionally, the study aimed to shed light on the available scientific evidence for this topic. 

## Materials and methods

The study was independently reviewed and approved by the Institutional Review Board (IRB) at King Saud University with research project number E-18-3126 and CDRC No: FR 0451 and complied with the rules related to the “Research Ethics on Living Organisms” issued by Royal Decree no. (M/29) and with the World Medical Association Declaration of Helsinki. Patients and accompanying persons including the general public provided verbal consent, which was approved and advised by the IRB. The consent was prepared in accordance with the WHO “Research Ethics Review Committee”. Each patient was asked to complete the survey while waiting in the patient waiting area. Confidentiality of participants’ information and privacy of data were maintained. 

We designed a cross-sectional survey with two questionnaires. The first questionnaire was distributed among the interns, clinicians, and faculty members of our institution and other distinguished dental faculty in the city of Riyadh, Kingdom of Saudi Arabia. This questionnaire was distributed in both printed and online forms. It included questions regarding demographic data and years of clinical experience. Short descriptive questions were used to determine the respondents’ opinions on coffee consumption, its relationship with the efficacy of dental local anesthesia, and the types of local anesthetic failures encountered.

The second questionnaire was prepared in Arabic and English languages in both printed and online forms to be distributed among patients and the public. Patients who reported to the outpatient and specialist clinics for routine dental appointments and the public accompanying the patients were approached to complete this questionnaire, which included questions regarding demographic data and the respondents’ experience and knowledge of coffee consumption and its effect on local anesthesia. The study variables included the effect of smoking, age, gender, drugs, other beverages taken by the patients, and the presence of any systemic diseases. None of the variables had missing data for > 1% of the responses. 

For the surveys, we included all patients and the public aged above 18 and less than 60 years, regardless of whether they consumed coffee (caffeinated/decaffeinated), and the general practitioners and specialists of all branches of dentistry. We excluded patients and healthy individuals aged below 18 years and above 60 years and pregnant females since the rate of caffeine metabolism vary largely in such cases [7]. 

The questionnaires were designed based on the Likert scale. A 5-point scale was used to assess agreement and frequency. Yes/no and open-ended questions were used for the assessment. The questionnaires were formulated by the authors and not copied or taken from other authors/institutes. The design of our survey instruments was informed by previously published surveys [14-15]. The instruments were pretested by five clinical investigators unaffiliated with the research team and were modified iteratively to improve clarity, face validity, and content validity. Content validity was improved by adding distinguished dental faculty who were practicing privately outside our institute as respondents to the survey. Face validity was adjusted by modifying certain questions on both the patient and the dentist survey forms, e.g., by modifying the question on the number of cups of coffee into one on coffee consumption in milligrams (mg). The sample size calculation was based on a well-established protocol [16]. 

The caffeine content in each cup calculation was done based on estimates reported in several studies and the United States (US) Food and Drug Administration (FDA) database, which has provided the caffeine content in beverages and drugs. These values when multiplied by the average size of one cup (240 ml for regular coffee and 25 ml for Arabic Gahwa) and the number of cups will give an estimate of the caffeine content consumed [17-18].

Data were analyzed with descriptive linear statistics using IBM Statistical Package for Social Sciences (SPSS) (IBM SPSS Statistics, Armonk, NY) software. The Chi-squared test was used to compare variables between both participant groups to check the relationship between dental local anesthetic failures among coffee drinkers and coffee non-drinkers. The Mann-Whitney U test and p-values were used to compare the relationship between the required number of dental local anesthetic doses to operating on coffee drinkers and coffee non-drinkers. In addition, the interquartile range (IQR) and median were compared using the Mann-Whitney U test and p-values obtained to identify statistically significant differences in the caffeine intake in mg between the dental local anesthetic failure and non-failure groups. P-values were also obtained to assess significant relationships between the beliefs of public participants and clinicians on the effect of coffee on dental local anesthesia.

## Results

Public survey results

We received 430 responses from the public; 82% of the public participants were young adults aged between 18 and 40 years, 67% were females, and 57% of the participants were educated. More than 40% of the public participants believed that the local anesthetic failure they experienced could be due to excessive coffee consumption. More than 50% of the participants consumed Arabic coffee and other beverages containing caffeine, like cola and tea, and 50% of the public participants consumed more than one cup of coffee daily. When we considered other caffeinated beverages, 40% responded that they consumed one cup daily.

In assessments of other patient-related factors that may have affected local anesthesia failure, more than 80% of the patients reported no smoking habit, no other relevant medical conditions, and no drug history. We found a statistically significant association between the level of education and the belief in local anesthesia failure due to caffeine consumption. More than 50% of the educated public believed that there was no association between the failure of local anesthesia and caffeine consumption. When considering the relationship between the numbers of cups of coffee consumed among those who experienced local anesthesia failure, those who consumed four or more cups experienced more failure. When the type of coffee was considered, no difference was noted. In assessments based on smoking status, 40% of smokers experienced local anesthesia failure. Most participants did not experience local anesthesia failure when other variables, like smoking, medical status, and drug intake, were considered (p < 0.01) (Table 1). Among coffee drinkers and non-coffee drinkers, these variables were potential components with a masking effect. 

**Figure 1:**
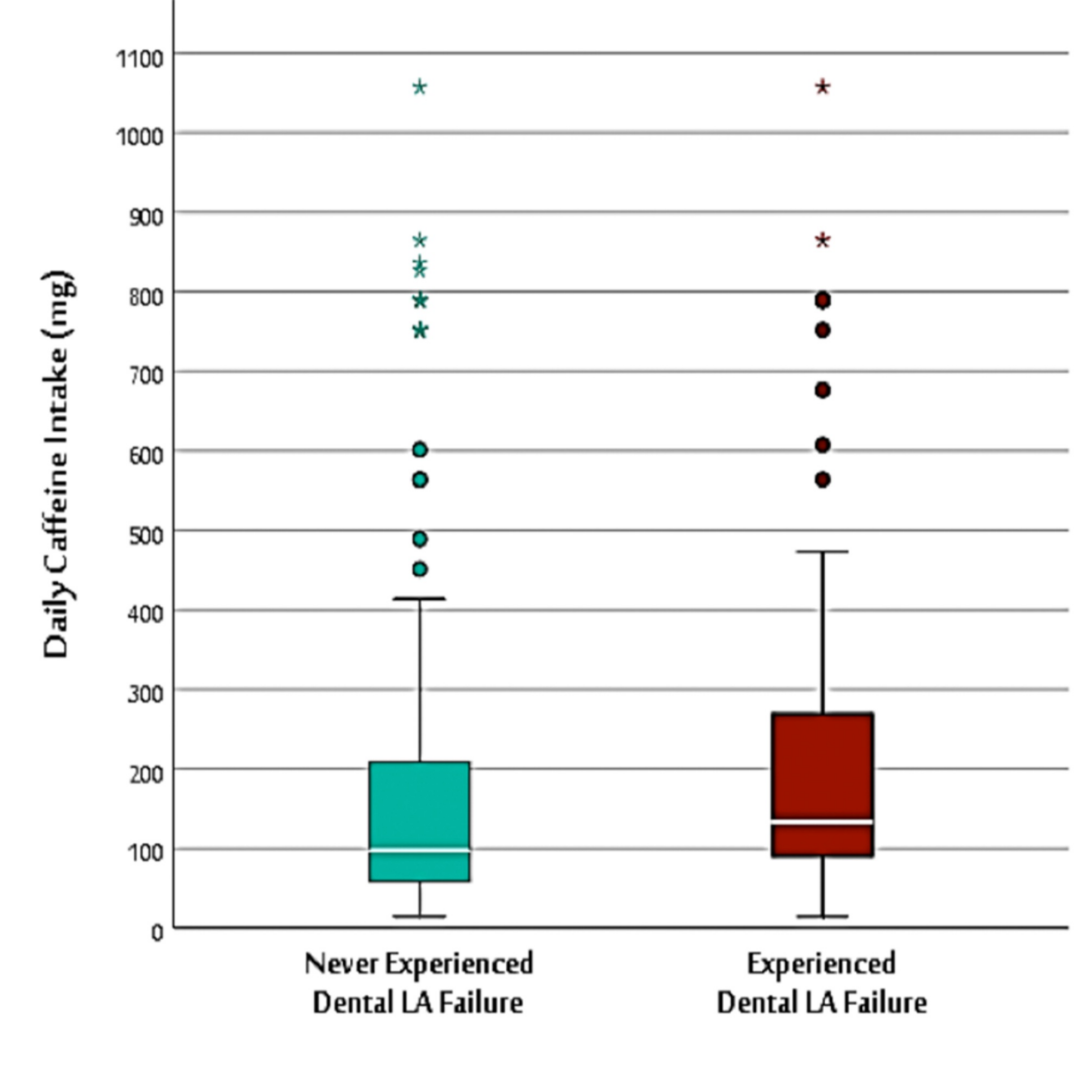
Box-and-whiskers plot of the distribution of daily caffeine intake among dental local anesthesia (LA) failure and non-failure groups The green box indicates the interquartile range (IQR) of the no-dental LA failure group, which shows a lower range of daily caffeine intake compared to the red box (IQR) of the dental LA failure group (Mann-Whitney U test p-value = 0.01**). The white lines in the middle of the boxes indicate the median, which is lower in the no-dental LA failure group (97.2 mg) compared to the dental LA failure group (133.4 mg) (median test p-value = 0.008**). The rounded dots (o) indicate mild outliers (> 1.5* IQR and  < 3* IQR), while the stars (*) indicate extreme outliers (> 3* IQR).

When considering the distribution of the number of dental local anesthesia doses, most patients required only one dose regardless of caffeine intake. Here, no statistical significance was shown in the Mann-Whitney U test. When considering the opinions on the effect of coffee on local anesthesia failure, only 25% of the public who experienced local anesthesia failure believed that the effect might be due to coffee (p < 0.01). The Mann-Whitney U test showed statistically significant p-values when daily intake of caffeine was considered for comparison between those who experienced local anesthesia failure and those who never experienced it. According to this test, the median caffeine intake was 97.2 mg (65 - 210 mg) and 133.4 mg (95 - 275 mg) in the no-failure and local anesthesia failure groups, respectively (p = 0.008) (Figure 1).

**Table 1:** The Relationship Between Dental LA Failure and Participants’ Medical and Drug History Stratified by Coffee Consumption for the Confounding Effects (*) Indicates statistical significance at 0.05. (**) Indicates a high statistical significance at a p-value of 0.01 (Chi-squared test was used to compare categorical variable groups) LA: local anesthesia

Variable		Coffee Drinkers						Coffee Non-Drinkers				
		Experienced Dental LA Failure		Never Experienced Dental LA Failure		P-value	Experienced Dental LA Failure			Never Experienced Dental LA Failure		P-value
		Count	Row %	Count	Row %		Count		Row %	Count	Row %	
Smoking status	Smoker	19	39%	30	61%	0.019*	4		50%	4	50%	0.074
	Non-smoker	61	23%	205	77%		21		22%	75	78%	
Medical conditions	Any chronic illness	23	44%	29	56%	0.001**	7		39%	11	61%	0.105
	No chronic illness	57	22%	207	78%		18		21%	68	79%	
Drugs reported for a possible effect on dental LA failure	Any drug	4	40%	6	60%	0.002**	1		50%	1	50%	0.511
	No drug reported	31	39%	49	61%		8		28%	21	72%	
	Not taking drugs	43	20%	177	80%		15		21%	57	79%	

Clinician survey results

**Figure 2:**
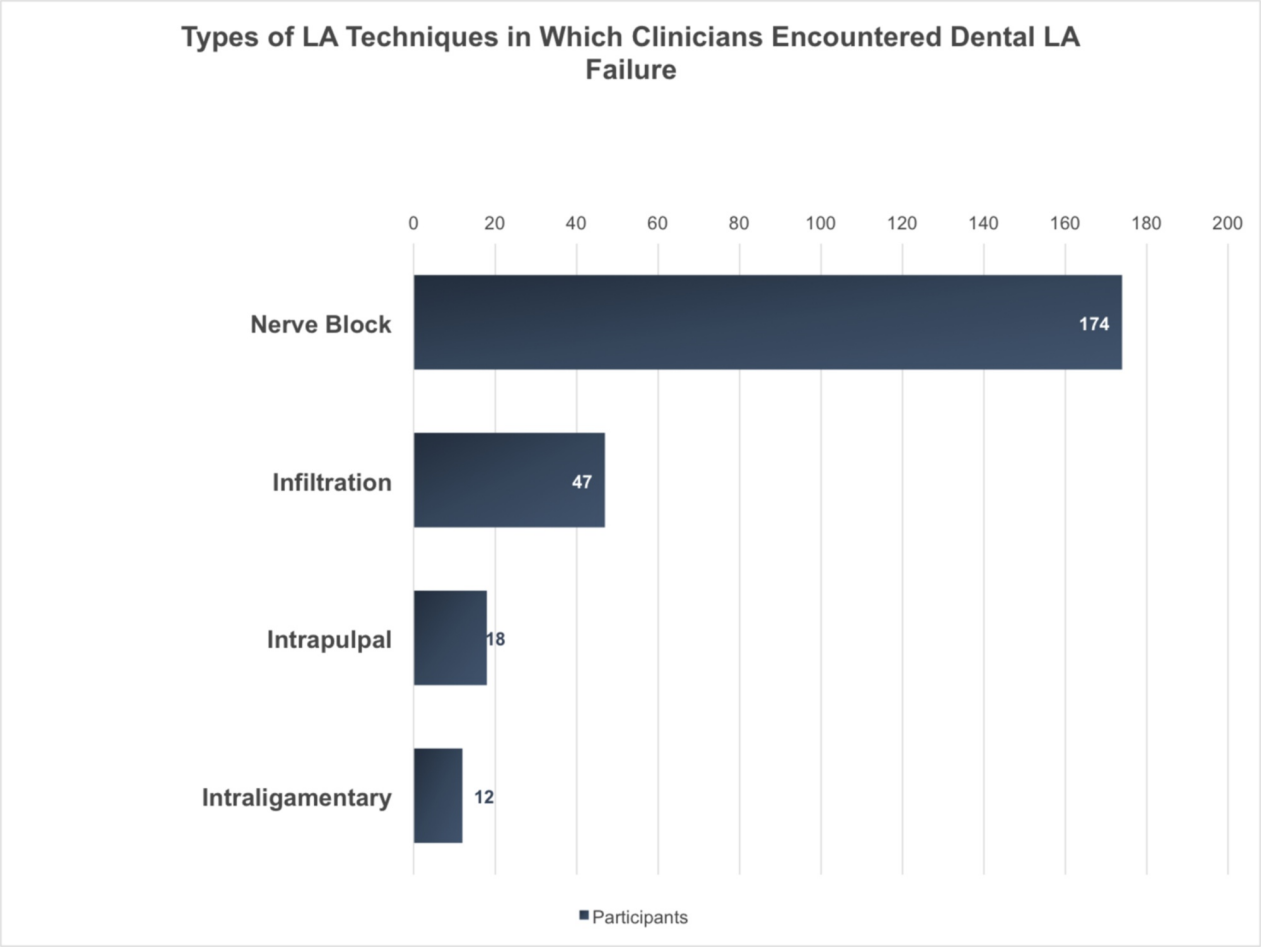
Types of LA techniques in which clinicians encountered dental LA failure LA: local anesthesia

**Figure 3:**
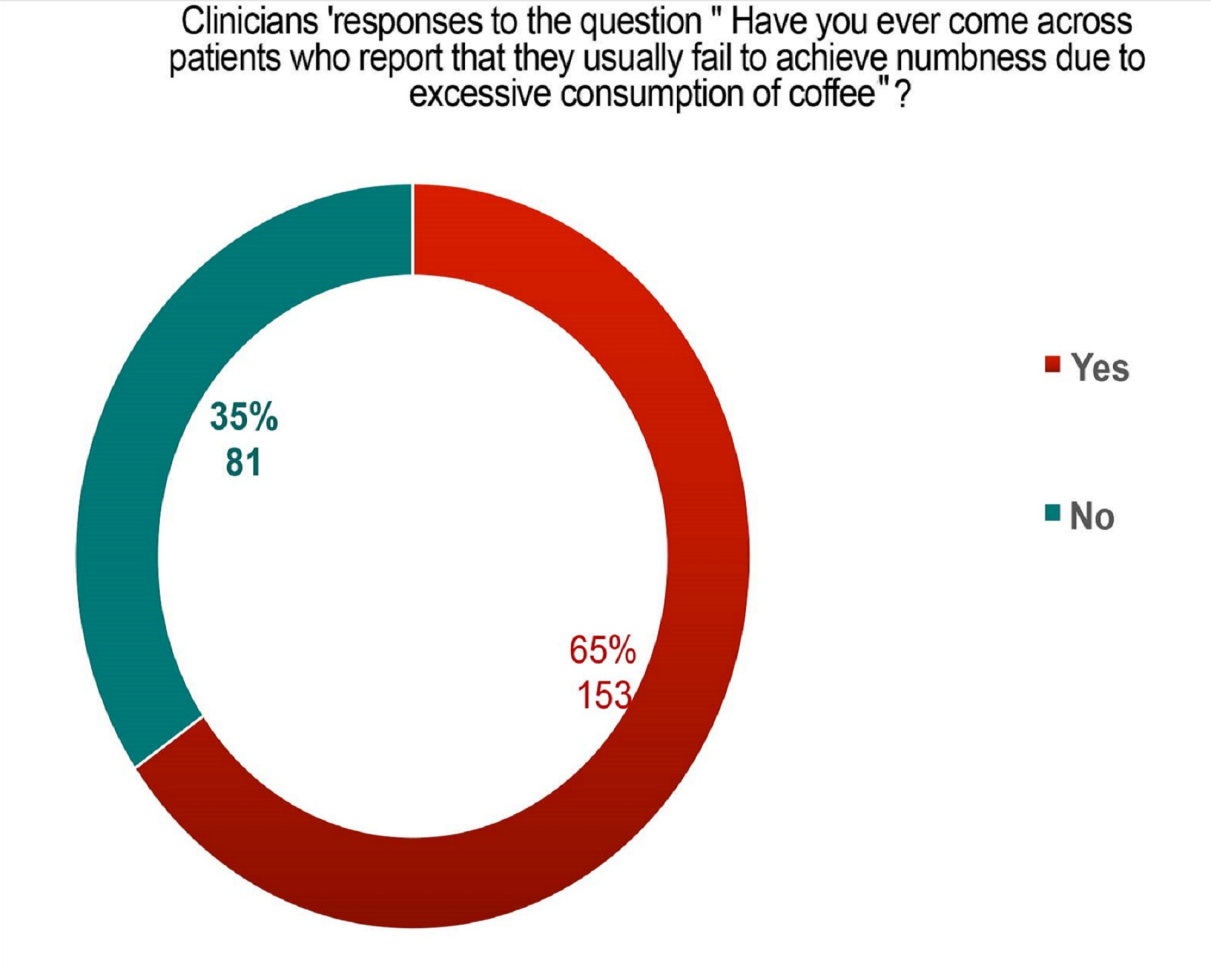
Clinicians’ responses to encountering patients with frequent dental LA failure due to excessive coffee consumption LA: local anesthesia

Among the 235 clinician participants, 50% had less than five years of clinical experience, 29% had more than 10 years of experience, and 37% were specialists (Table 2). Among the clinicians, 55% used mepivacaine 3%, as a local anesthetic in dental practice and 40% used xylocaine. More than 50% of the clinicians had experienced local anesthesia failure; 65% of the clinicians reported that they encountered patients with local anesthesia failure, and they thought it could be due to excessive coffee consumption (Figure 2). When assessing the opinions of clinicians on the effect of coffee on dental local anesthesia failure, 31% agreed on the positive effect of coffee on local anesthesia failure. Among these, only 9% were aware of the scientific evidence for the effect of coffee consumption on local anesthesia failure. In assessments for the type of local anesthesia technique used during which clinicians encountered failure, 74% reported failure of nerve block and 20% reported failure during infiltration (Figure 3). According to the clinicians’ responses, 61% required higher dental local anesthesia doses to achieve numbness. According to them, an average of two doses were required to achieve nerve block anesthesia. However, 81% of the clinicians were of the opinion that no medications or beverages interfere with local anesthesia efficiency. 

**Table 2:** General Characteristics of Study Participants in the Clinicians’ Survey (n = 235 Participants) The Chi-squared test was used to compare categorical variables groups. LA: local anesthesia

		Frequency	Percentage	P-Value (Believes in the Effect of Coffee on Dental LA Failure)
Years of Clinical Experience	0 - 5 years	117	50%	0.111
	6 - 10 years	50	21%	
	11 - 15 years	28	12%	
	More than 15 years	40	17%	
Level of Education	Intern	58	25%	0.238
	General practitioner	44	19%	
	Postgraduate	44	19%	
	Specialist	84	37%	

**Figure 4:**
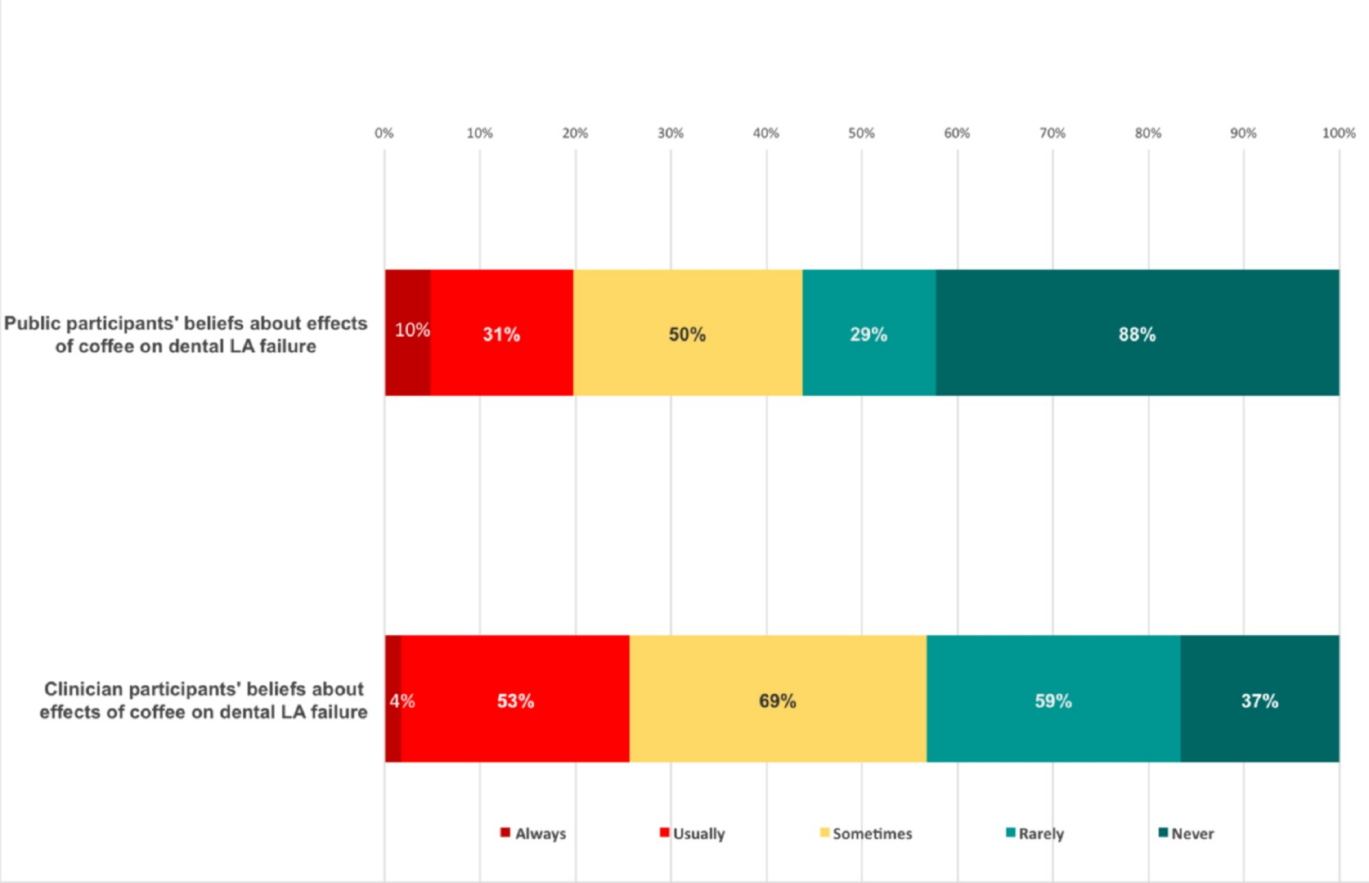
Public's and clinicians' beliefs on the effects of coffee on dental anaesthesia The Chi-squared test p-value was < 0.001, which indicates highly significant differences between the public's and the clinicians’ beliefs. The median category for public participants was those who think that coffee rarely causes dental LA failure, while the median category for clinicians was those who think coffee sometimes causes dental LA failure. LA: local anesthesia

When we consider variables like dentist experience, no significant association was found between clinician experience and the presence of dental LA failure. Finally, we found a statistically significant difference in the opinion between the public and the clinician, when the median category was taken into consideration (Figure 4).

## Discussion

In routine clinical practice, many patients report a history of failure of local anesthesia, which they believed was due to the excessive consumption of coffee. Due to the lack of scientific evidence for this assumption, this study aimed to assess the experience, knowledge, and awareness of coffee consumption and its effect on local anesthesia among the public and the clinicians. Caffeine, which is the active coffee component and one of the most extensively studied food components, is a psychoactive drug widely consumed in the form of beverages. Although there are numerous published articles regarding the pharmacokinetics of caffeine and its effects on health, we did not come across any study that directly relates to the effect of caffeine on local anesthetic action. However, an interesting study by Alobaid et al. to assess the effect of caffeine on the onset, efficiency, and duration among female patients was found. Here, the authors found increased failure rates and delayed onset of LA among high caffeine intake groups and concluded that the myth of caffeine effect on the onset, duration, and efficacy of local anesthesia was not proven [19]. However, when we surveyed clinician knowledge and beliefs, we found that 31% agreed on the positive effect of coffee on local anesthesia failure. Among these, only 9% were aware of the scientific evidence for the effect of coffee consumption on local anesthesia failure. The only available evidence is from a study showing that caffeine reverses the effects of general anaesthesia [20]. On the other hand, there is enough data in the literature showing that caffeine may interact unfavorably with sedative/anesthetic drugs to trigger a particularly aggressive apoptotic response in the infant's brain [21]. 

On reviewing the literature to find scientific evidence, we primarily looked at studies dealing with the failure of local anesthesia, after which we looked at the mechanism of action of the active component in coffee, i.e., caffeine. Variable factors have been reported to contribute to the failure of local anesthesia. They may be either patient-related, such as anatomical, pathological, and psychological factors, or operator-dependent, like the choice of technique and amount of solution used [10, 12]. Evidence shows that highly stressed or anxious patients are poorer candidates for surgery under local anesthesia. Adrenaline secreted in response to fear or pain can delay the onset, anaesthesia is not pronounced enough, or it may wear off too quickly [22]. Studies have shown that anxiety will be relatively higher before dental surgery, and patients with greater anxiety in the treatment environment feel more pain; thus, it is important to reduce anxiety to alleviate pain during treatment [23]. Therefore, patient psychology may also contribute to local anesthesia failure. 

Studies covering the action of caffeine have shown that it increases the plasmatic levels of stress hormones, including catecholamines such as adrenaline and cortisol [8]. Moreover, chronic consumption of caffeine causes a tolerance to its adenosine receptor-dependent effects, inducing upregulation of the receptors, which is characterized by headache, anxiety, and flushing [8]. This could be one of the reasons for inadequate anesthesia in this category of patients. One can infer that due to stress, the anxiety and alertness provoked by caffeine could lead to failure of local anesthesia. 

On the other hand, we also found interesting studies on the action of caffeine on the pain pathway. Caffeine causes most of its biological effects by antagonizing all types of adenosine receptors. Adenosine is an endogenous substance in the CNS and can exert its action by activation of adenosine A1, A2a, A2b, and A3 receptors on the cell surface [24]. Adenosine has a dual nature in that it is a molecule that can cause and relieve pain via different adenosine receptors [25]. Long recognized as an analgesic when acting through A1 receptors, which are central receptors, adenosine also contributes to chronic pain through its A2b receptors, which are peripheral receptors. In summary, caffeine opposes the analgesic effect if it acts centrally on A1 receptors [25]. However, this can be related to the effect of local anesthesia. Local anesthetics inhibit the sensation of pain by interrupting neural conduction via the prevention of action potential propagation. This process occurs via a blockade of the influx of sodium ions into the cells through their channels, which causes a change in voltage and prevents the transient increase in permeability of the nerve membrane, thereby preventing depolarization and action potentials and thus stopping the initiation of the pain pathway [26]. Therefore, caffeine can oppose the analgesic action of adenosine receptors, and it can oppose the action of local anesthesia via the same mechanism. Although there is no evidence showing that local anesthesia and caffeine act on the same receptors, the synergistic actions of different analgesic medications (e.g., paracetamol and nonsteroidal anti-inflammatory drugs (NSAIDs)) on different receptors and pathways also eventually culminate in a synergistic effect centrally. Another study that may support this argument was a Cochrane review showing that caffeine, when used as an adjuvant (along with analgesics like paracetamol and ibuprofen), had a small but statistically significant benefit at doses of 100 mg or more [27]. The other supporting evidence is from studies in premature infants showing that caffeine may augment the neurotoxic action of anesthetic medication indicated for neonatal sedation/anesthesia in the neonatal intensive care unit (NICU) by triggering widespread apoptosis in the developing brains of premature infants [21]. 

Although no significant association was found when comparing the clinicians' experience and dental local anesthesia failure, we found a highly significant difference in the opinions of the public and the clinicians when the median category was taken into consideration. The median category for public participants consisted of those who did not think that coffee caused dental local anesthesia failure, whereas the median category for clinicians consisted of those who thought that coffee sometimes caused dental local anesthesia failure. This difference could be explained by the experiences of dentists who frequently had trouble and failure of local anesthesia, especially nerve blocks, during their daily practice. We found that 57% of the clinicians had often experienced the failure of local anesthesia. Therefore, it would be logical for them to at least consider coffee as a potential cause of failure of local anesthesia. In terms of percentages, 31% believed that coffee had an effect, while 38% neither agreed nor refused the statement. However, only 8.7% were aware of the scientific basis for the relationship between coffee and anesthesia. 

In addition, in assessments of caffeine consumption in mg, a significant association was found between the amount consumed and the experience of the failure of local anesthesia. The results showed a higher caffeine content of 133.4 mg among participants who experienced dental LA failure. Adults over the age of 25 years have an estimated consumption of approximately 2.4 mg/kg/day. In the literature, the average daily consumption of caffeine varied depending on the survey, year conducted, and the sources considered [28]. However, it has most recently (i.e., 2011 - 2012) been reported as 142 mg per day for adults and children in the US, a decrease from previous years (e.g., the average consumption of 175 mg/day in 1999 - 2000), which is largely attributed to a reduction in soda consumption. Caffeine is known to have generally dose-dependent effects with positive or desirable effects at lower doses (i.e., ≤ 400 mg) and undesirable effects generally above this level of intake [28]. 

When considering other patient-related factors, like smoking status, medical condition, and drug intake, which may affect local anesthesia failure, 80% showed no smoking habit and no other relevant medical conditions or drug intake. Therefore, these variables are potential components that may have a masking effect since 40% of the smokers experienced local anesthesia failure. It has been documented that both nicotine and caffeine have stimulant effects, but smoking increases the clearance of caffeine because of its action on cytochrome P450 (CYP1A2) [29-30]. Thus, smoking may minimize the effect of caffeine on adenosine receptors and, therefore, the action of local anesthesia is not weakened or opposed.

## Conclusions

In conclusion, surprisingly, the majority of clinicians believed that caffeine had an effect on the reduction of local anesthesia; however, only a few of them held scientific knowledge. The available scientific evidence relates that caffeine can influence cognitive performance by increasing alertness, as well as sleep deprivation and causing stress and anxiety, which partially explains the local anesthetic failure among coffee consumers. This study was confined to a small area and sample size. A similar multicenter evaluation would enable us to draw conclusions that are more definitive.
